# Biological costs and benefits of selective breeding for disease resistance using marker-assisted or field-based selective breeding in honey bees (*Apis mellifera*)

**DOI:** 10.1093/jee/toag044

**Published:** 2026-03-03

**Authors:** Robert W Currie, Lynae P Ovinge, Derek Micholson, Abdullah Ibrahim, Michael Peirson, Heather Higo, Elizabeth Huxter, Maria Marta Guarna, Leonard J Foster, Stephen F Pernal, Shelley E Hoover

**Affiliations:** Department of Entomology, University of Manitoba, Winnipeg, MB, Canada; Department of Biological Sciences, University of Lethbridge, Lethbridge, AB, Canada; Department of Entomology, University of Manitoba, Winnipeg, MB, Canada; Agriculture and Agri-Food Canada, Beaverlodge Research Farm, Beaverlodge, Alberta, Canada; Agriculture and Agri-Food Canada, Beaverlodge Research Farm, Beaverlodge, Alberta, Canada; Department of Biochemistry and Molecular Biology, Michael Smith Laboratories, University of British Columbia, Vancouver, BC, Canada; Department of Biochemistry and Molecular Biology, Michael Smith Laboratories, University of British Columbia, Vancouver, BC, Canada; Department of Biochemistry and Molecular Biology, Michael Smith Laboratories, University of British Columbia, Vancouver, BC, Canada; Department of Biochemistry and Molecular Biology, Michael Smith Laboratories, University of British Columbia, Vancouver, BC, Canada; Agriculture and Agri-Food Canada, Beaverlodge Research Farm, Beaverlodge, Alberta, Canada; Department of Biological Sciences, University of Lethbridge, Lethbridge, AB, Canada

**Keywords:** honey bee, marker-assisted selection, integrated pest management, hygienic behavior, social immunity

## Abstract

Honey bee (*Apis mellifera* L.) breeders seek to enhance disease resistance by selecting for behavioral resistance traits such as hygienic behavior and *Varroa*-sensitive hygiene. However, traditional phenotypic assays for these traits are labor-intensive, limiting scalability. Antennal protein biomarkers correlated with these behaviors offer a promising tool for marker-assisted selection (MAS), potentially increasing phenotyping efficiency and accelerating genetic gains. This study evaluated the performance of honey bee colonies from 4 stocks: those selected using field-based phenotypic assays (FAS) or MAS, versus unselected domestic stock (BEN) or imported commercial (IMP) stock. Colonies were situated across 3 distinct geographic regions and were managed with or without acaricide treatment for the treatment of *Varroa destructor* mites to assess both the benefits of resistance under parasitic stress and potential fitness costs under low-stress conditions. Across regions, MAS and FAS stocks performed comparably in productivity and pathogen metrics and were equal to or more productive than the unselected BEN and IMP stocks. At specific locations and time points, MAS and FAS colonies maintained significantly lower *Varroa destructor* populations in the absence of acaricides, demonstrating enhanced natural mite suppression. Selection for hygienic behavior was positively associated with colony health and fitness, with no evidence of biologically meaningful trade-offs. Increased overwinter food consumption in selected colonies reflected larger fall populations rather than a direct cost of resistance.

## Introduction

The health of honey bee colonies (*Apis mellifera* L. (Hymenoptera: Apidae)) is increasingly threatened by a growing range of parasites, viruses, and pathogenic microorganisms. The globalized nature of modern apiculture facilitates both the transmission and geographic spread of these stressors. In Canada, compromised honey bee health is most often observed as elevated overwinter colony mortality, which has averaged 28.4% between 2007 and 2025 (CAPA 2025).

According to the principles of integrated pest management, multiple approaches should be employed in combating pathogens and parasites while minimizing negative aspects associated with control, such as the development of resistance or exposure of food products to pesticide residue. Among the foundational preventative tactics is selective breeding for resistance or tolerance traits against pathogens. In addition to individual immune responses, honey bees exhibit collective behavioral defences known as social immunity; these cooperative behaviors among colony members help prevent or mitigate disease transmission (Cremer et al. 2007). These behaviors include worker activities such as grooming or the coordinated removal of compromised brood. These behaviors provide insect societies with an added layer of disease prevention or mitigation that arises from behaviors with workers cooperating as a group to complete a task or individuals interacting towards one another as in grooming behavior.

One of the best-characterized social immunity traits is hygienic behavior, in which worker bees detect and remove diseased or dead brood from the colony. Hygienic behavior provides protection against brood pathogens such as *Paenibacillus larvae* (Genersch et al. 2006), the causative agent of American foulbrood, by interrupting the development of the pathogen before it reaches the spore stage (Woodrow and Holst 1942, Rothenbuhler 1964, Spivak and Gilliam 1998, Spivak and Danka 2021). A related trait, Varroa-sensitive hygiene, refers to the ability of workers to detect and remove brood infested with *Varroa destructor* mites (Ibrahim and Spivak 2006, Harris 2007). Both behaviors occur naturally within honey bee populations but can be enhanced through selective breeding (eg Ibrahim et al. 2007, Guarna et al. 2017, Maucourt et al. 2021, Avalos et al. 2024). As with immune defences in individuals, social immunity traits are presumed to incur fitness costs or life history trade-offs; however, such costs are not always observed and remain poorly studied (Rivera-Marchand et al. 2008, Labbé et al. 2010, Meunier 2015, Leclercq et al. 2017, Cremer et al. 2018).

Breeding honey bees for trait improvement presents considerable challenges compared to other livestock. Honey bees may be prone to inbreeding depression (eg Bienefeld et al. 1989), and successful mating requires queens to copulate with multiple drones during flight under specific environmental conditions (Oxley and Oldroyd 2010). Further complicating selection is the difficulty of identifying colony-level phenotypes, especially for traits like hygienic behavior or *Varroa*-sensitive hygiene. Phenotypic assays, such as freeze-killed or pin-killed brood tests, are time-consuming, may require specialized materials (eg liquid nitrogen), and are sensitive to both management practices and environmental variability. Newer assays, such as the unhealthy brood odour (UBeeO) test, which is based on pheromones emitted by diseased brood, show promise but still rely on labor-intensive behavioral observation (Wagoner et al. 2020). Similarly, field-based evaluations of *Varroa*-sensitive hygiene, though effective, are also prohibitively tedious for routine use (Spivak and Danka 2021). To advance commercial bee breeding, more efficient phenotyping strategies are needed.

Marker-assisted selection (MAS) using proteomic biomarkers offers a promising alternative. A foundational study identified 7 antennal proteins in worker bees that were predictive of hygienic behavior and associated with reduced *Varroa* infestation (Guarna et al. 2015). An expanded protein selection panel was then developed by identifying additional 6 proteins, 4 of which were correlated with *Varroa*-sensitive hygiene (Guarna et al. 2017). These biomarkers were used to guide selective breeding of a MAS stock over 3 generations. Simultaneously, a field-assisted selection (FAS) stock was selected for hygienic behavior using freeze-killed brood assays. After 3 generations, MAS-selected colonies exhibited superior hygienic behavior compared to an unselected benchmark and showed improved survival when challenged with bacterial or parasitic infections, comparable to FAS-selected colonies. Further details on breeding protocols and experimental validation of these lines are provided in Guarna et al. (2017) and Bixby et al. (2017).

The objective of this study was to evaluate the stocks ­selectively bred for improved hygienic brood removal using MAS and FAS approaches in Guarna et al. (2017) and compare them against unselected IMP and domestic stocks. Colony ­performance was assessed across a range of parasite and pathogen challenges, while also examining potential fitness costs using metrics such as brood production, adult population size, colony weight, and honey yield. Colonies were exposed to 2 levels of *Varroa* mite pressure to evaluate interactive effects of stress and hygienic behavior. We assess performance across an array of parasite and pathogen challenges, while also evaluating potential fitness costs using colony-level metrics such as brood production, adult population size, colony weight, and honey yield. By investigating both the benefits and potential trade-offs associated with resistance traits, this study aims to inform the practical value and adoption potential of MAS- and FAS-bred honey bees in commercial apiculture, and livestock breeding more broadly.

## Methods

### Stock

Guarna et al. (2017) describes the selective breeding program used to increase hygienic behavior in honey bees using both protein markers and field-based assays. Here, we report field experiments comparing gains in hygienic behavior after 3 generations using these stocks, selected using MAS versus field assisted selection (FAS), relative to domestic benchmark (BEN) and IMP stocks. We assessed the benefits and costs of selection by measuring parasite and pathogen resistance, as well as colony growth and productivity. F3 Benchmark queens were produced by a single queen producer in British Columbia and were derived from western Canadian production colonies, while IMP queens were obtained from New Zealand. Due to the large number of queens required, MAS and FAS queens were naturally mated by a single producer in isolated mating apiaries (>19 km from another apiary) with selected drones, whereas imported queens were mated with unselected colonies in their country of origin. MAS virgin queens were reared from 11 queen mother colonies and mated with drones from 7 source colonies. Similarly, FAS virgin queens were reared from 10 queen mother colonies and mated with drones from 12 source colonies.

To standardize starting conditions for the experiment, honey bee packages (1 kg of worker bees plus a queen) were IMP from New Zealand and installed in single-deep 10-frame Langstroth hives across 3 locations in Canada: Lethbridge, Alberta (48 colonies, 1 apiary, 49°41′24.7″N, 112°46′40.0″W), Beaverlodge, Alberta (92 colonies, 2 apiaries, 55°11′25.3″N, 119°18′14.6″W (*n* = 48), and 55°11′10.6″N, 119°17′49.4″W (*n* = 44)), and Glenlea, Manitoba (92 colonies, 2 apiaries, 49°38′44.05″N, 97°7′20.21″W (*n* = 48)), 49°38′44.80″N, 97° 9′6.51″W (*n* = 44)). Colonies were established on 13 May 2013 in Alberta and on 17 May 2013 in Manitoba. The original queens were removed from all packages, and paint-marked experimental queens were then introduced into each queenless package on 16 May in Alberta and on 17 May in Manitoba. Colonies headed by queens from the 4 stocks (MAS, FAS, BEN, and IMP) were evenly distributed within apiaries and arranged in randomized blocks of 4, with entrance orientations rotated to minimize drifting effects. All colonies were maintained in single brood chambers, with honey supers added above a queen excluder as needed.

To assess stock performance with and without *Varroa* mite pressure, half of the colonies of each stock at each site were randomly assigned to receive acaricide treatment in spring and fall, while the remaining colonies were left untreated. Apivar (active ingredient amitraz) was applied at colony establishment in spring, with strips removed after 21 to 22 d, and again in September, with strips removed after 40 to 44 d, consistent with label directions. At the time of the study, there was no evidence of Amitraz resistance at any of the study locations.

### General Colony Management

At establishment, all colonies were fed a commercial 0.45 kg pollen patty (15% irradiated pollen) and, consistent with industry practices, treated for *Vairimorpha* (formerly *Nosema*) spp. with Fumagilin-B (DIN 02231180) (2.38 g in 2.0 liter 1:1 sucrose syrup) in frame feeders, followed by a second treatment 1 wk later. In Manitoba, colonies were also treated with oxytetracycline (DIN 02231111) for the treatment of American Foulbrood according to label directions. In September, all colonies received a fall treatment of Fumagilin-B (4.76 g in 2.0 liter of 2:1 sucrose syrup) applied over 2 successive feedings.

Colonies were inspected regularly, and supersedure cells were removed when the experimental queen was present. Colonies that lost their experimental queen through death or supersedure were removed from the experiment, and data from those colonies were excluded from analyses from the last confirmed observation onward (see Supplementary Table S2 for the number of colonies remaining in the experiment at each assessment date). All colonies were wintered indoors under standard conditions (constant darkness, 4 °C, and controlled ventilation) starting late October or early November 2013 until early April 2014 (Currie et al. 2015).

### Colony Assessments

Experimental colonies were assessed at 6 timepoints: 13 to 17 May (establishment), 2 to 9 July, 19 to 29 August, 18 to 24 October 2013, and 9 to 15 April and May 2014. The first assessment coincided with approximately one worker population turnover (∼61 d; average period as brood 21 d (Winston 1987), plus mean adult survival 40 d (Sakagami and Fukuda 1968)). At each assessment, the colonies were: (i) visually inspected for disease symptoms and queen presence, (ii) adult bee samples were collected to estimate pathogen abundance, and (iii) and colony population was estimated using seasonally appropriate methods (photography in July, August, and May; cluster scores in October and April, see below for assessment details).

Disease inspections included examination of brood for American foulbrood, European foulbrood, chalkbrood, and sacbrood, and adult bees for deformed wing virus. Disease incidence was scored as none (0 infected larvae, pupae, or adult bees), low (1 to 10 infected), medium (11 to 100), or high (>100 infected), but due to low overall incidence, diseases were analysed as present (>1 infected) or absent (0 infected larvae, pupae, or adult bees) at the colony level.

### Colony Population Assessments

Adult bee and sealed brood populations were estimated using a photographic method during summer assessments (July and August 2013; May 2014). Before morning flight, each frame containing adult bees was photographed on both sides in a shaded photo box, and remaining bees on hive surfaces were visually estimated and added to colony totals (as previously described in Peirson et al. 2024). Frames containing sealed brood were photographed later the same day, after adult bees were brushed off. Images were analyzed using Honeybee Complete 4.2 to estimate adult bee numbers and sealed brood cells (WSC Scientific GmbH, Heidelberg, Germany).

During colder assessment periods (Oct 2013 and April 2014), colony populations were estimated using cluster scores (Nasr et al. 1990). Colonies were assessed on mornings when it was cold enough that the bees were not yet readily flying. To estimate the cluster size, colonies were examined from the top and the bottom of the brood chamber to estimate the number of frame spaces filled with bees. The number of inter-frame spaces laterally filled with bees was estimated to the nearest 0.25. The cluster score was calculated for each colony as the average of the top cluster score and the bottom cluster score. Colony survival was assessed using logistic analyses (SAS Proc CATMOD).

### Pathogen Abundance

At each assessment, one sample of ∼300 adult bees was taken from brood frames, preserved in 70% ethanol, and used to estimate *Varroa* abundance and colony American foulbrood infection. A second sample of ∼100 adult bees collected from honey frames was preserved to estimate *Vairimorpha* spp. infection levels and prevalence. *Varroa* abundance was measured by shaking the 300-bee sample in a screened container with 70% ethanol until all the mites were dislodged and counted (Gatien and Currie 2003). Abundance was then expressed as the number of *Varroa* per 100 bees.

Spore concentrations of *P. larvae* in adult bees were estimated using microbiological techniques adapted from Pernal and Melathopoulos (2006). After washing the 300-bee sample in ethanol, 120 bees were placed in a stomacher bag and resuspended in 30 ml 1× PBS buffer and homogenized for 1 min at medium speed (Seward Stomacher 80 Biomaster, Seward Laboratory Systems Inc., Davie, Florida, United States). Two 1 ml aliquots were centrifuged at 6708 × *g* for 5 min, the supernatant reduced to 100 µl, and the pellets were resuspended in 900 µl PBS. One aliquot was used immediately, and the other stored at –20 °C as a reference. Suspensions were pasteurized at 85 °C for 15 min to kill non-spore-forming organisms. Five 20 µl replicate drops of each suspension were directly pipetted onto separate sections of a plate containing modified *Paenibacillus larvae* agar (PLA) media and incubated at 34 °C and 5% CO_2_ (de Graaf et al. 2006). The number of *P. larvae* CFU on each plate section was determined at day 7; any plates having more than 30 colonies per drop were uncountable and had their original suspensions serially diluted in PBS and re-plated. Bacterial colonies were confirmed as *P. larvae* by their morphology and growth rate and by their inability to break down 5% hydrogen peroxide (catalase negative) (Haynes 1972).

To assess *Vairimorpha* infections at the colony level, abdomens from 60 adult bees were placed into 70% ethanol (1 ml per bee) and macerated for 1 min at medium speed (Seward Stomacher 80 Biomaster, Seward Laboratory Systems Inc., Davie, Florida, United States). A 6 μl aliquot of the macerate was loaded onto a Helber Z30000 counting chamber (Hawksley, Lancing, United Kingdom), and spores were counted under phase-contrast microscopy at 400× following Cantwell (1970). Remaining macerate was stored at –20 °C in 1.5 ml microcentrifuge tubes. To identify *Vairimorpha ceranae* and *V. apis*, the crude macerate was vortexed, and 200–400 μl was centrifuged to remove ethanol. DNA was extracted using the DNeasy Blood & Tissue Kit (Qiagen, Valencia, California, United States) and quantified spectrophotometrically (NanoDrop 2000C, Thermo Scientific, West Palm Beach, Florida, United States). PCR was performed on 50 to 100 ng of DNA using a multiplex system that co-amplified the 16S rRNA gene of *V. apis* and *V. ceranae* (Martín-Hernández et al. 2007) along with the honey bee ribosomal protein RpS5 gene (Thompson et al. 2007). PCR protocols follow van den Heever et al. (2016, see Supplementary Table S1).

### Honey Production

Honey production was assessed by weighing supers prior to placing them on colonies and following their removal from the hive. Each super contained 6 frames of fully drawn comb and 3 new frames of undrawn foundation, with additional supers added as needed to maintain a minimum of 2 supers per colony over the summer. Total honey production per colony was calculated by summing the net weights of all supers. Honey yield was evaluated for 191 experimental colonies across the 5 apiaries across 3 locations, however, only colonies that retained their experimental queen all summer were included in the analyses (*n* = 169 colonies).

### Hygienic Behavior Assay

In early autumn, after the nectar flow ended and all honey supers were removed, hygienic behavior was assessed in remaining colonies using a freeze-killed brood assay (as described for selective breeding of these stocks in Guarna et al. 2017). The test was conducted twice, approximately 1 wk apart: 29 August and 4 September in Lethbridge, 4 to 5 September and 9 to 10 September in Beaverlodge, and 3 September and 11 September in Manitoba. Equipment and procedures were identical across locations, and assays were timed to ensure similar colony populations and brood availability. Minor environmental variation was expected due to distances of 900 to 1,800 km between locations.

To perform this test, a frame of sealed brood containing solid patches of pink-eyed pupae was removed from each hive. Two brood patches were frozen by pressing 2 15 cm lengths of 50 mm diameter PVC pipe into each patch and pouring ∼300 ml of liquid nitrogen into each tube. After thawing, frames were returned to their colonies and removed 24 h later to assess hygienic behavior. The proportion of frozen cells fully uncapped and cleared of pupae was calculated relative to the total number of frozen cells (73 cells per tube).

### Statistical Methods

Statistical analyses were performed using SAS 9.4 (SAS Institute, Cary, North Carolina). Data for brood production, adult bees, cluster size, *Varroa* abundance, American foulbrood, and *Vairimorpha* spores, were analyzed by a mixed model ANOVA (Proc Mixed) with stock, acaricide treatment, and apiary site or location as main factors (fixed effects) in the model using a repeated measures design where colonies were treated as subjects (a random effect) and assessment date was a repeated measure. Where no variation among apiary sites was noted, data were grouped based on location. Data for American foulbrood spores and *Vairimorpha* spores were log-transformed, and *Varroa* abundance and hygienic brood removal proportions were arcsine-transformed prior to analysis. The full model was tested using an unstructured covariance structure (based on the lowest AIC values) using the ddfm = Kenward-Rogers option (Proc Mixed) when assumptions of homogeneity of variance were not met. Where significant interactions were found, single degree of freedom contrasts were examined using the SLICE option in SAS (*P* < 0.05). Where significant interactions occurred with season, further analyses were conducted for each individual assessment date. Data for hygienic brood removal, honey production, fall colony weight and weight loss over winter, which were sampled on each colony only once on one date, were analyzed by a mixed model ANOVA (Proc Mixed) with stock, acaricide treatment, and apiary site or location as main factors (fixed effects) in the model.

## Results

### Brood Production

When analyzed across all assessment dates, there were significant differences in overall brood production that were affected by both acaricide treatment (*F *= 11.80; df = 1, 153; *P *= 0.0008) and stock (*F *= 6.79; df = 3, 151; *P *= 0.0003) (Fig. 1). However, there was also a significant interaction among acaricide treatment, stock, and assessment date (*F *= 4.17; df = 6, 169; *P *= 0.0006); therefore, separate analyses were performed for each assessment date. Spring acaricide treatment increased overall brood production in July (*F *= 4.46; df = 1, 172; *P *= 0.036, Fig. 1A), August (*F *= 8.77; df = 1, 135 *P *= 0.004, Fig. 1C), and in the following May (*F *= 4.40; df = 1, 68; *P *= 0.04, Fig. 1E) relative to untreated colonies. In July (*F *= 4.11; df = 3, 172; *P *= 0.008, Fig. 1B) and August (*F *= 4.36; df = 1, 136; *P *= 0.006, Fig. 1D) the level of brood production was also affected by stock. In both months, both the FAS and MAS selected colonies had greater brood production than the benchmark colonies (*P* < 0.05; Tukey) but not the imported colonies (*P *> 0.05; Tukey). At the July and August assessments, there were no interactions between acaricide treatment and stock in their effect on brood production (*F *= 0.58; df = 12, 145; *P *= 0.86).

The overall effect of stock on brood production the following May (2014) also showed that both FAS and MAS stocks had greater brood production than the IMP stock, but not the BEN stock (Fig. 1E). However, the response to stock depended upon whether colonies had been previously treated with acaricide or not (*F *= 3.71; df = 3, 68; *P *= 0.016). Importantly, colonies that had received previous acaricide treatment showed no differences among stock in brood production (*F *= 1.27; df = 3, 68; *P *= 0.29; Slice), whereas brood production was affected by stock when acaricide treatment was absent (*F *= 5.61; df = 3, 68; P = 0.0017; Slice). Importantly, contrasts showed that only the IMP (*F *= 7.4; df = 1, 68; *P *= 0.008; Slice) and BEN stocks (*F *= 5.76; df = 1, 68; *P *= 0.019; Slice) showed greater spring brood production when exposed to acaricide treatments than when not treated (Fig. 1E). Overall brood production was greater in Manitoba than in Beaverlodge or Lethbridge on all 3 dates when it was assessed (*P* < 0.05 Tukey). Brood rearing in Beaverlodge and Lethbridge did not differ except for on the first assessment date at when the mean number of brood cells (± standard error) was higher in Lethbridge (9,276 ± 376 cells) than in Beaverlodge (8,179 ± 274 cells) while Manitoba had the highest (14,844 ± 287 cells) (Tukey <0.05).

**Figure:**
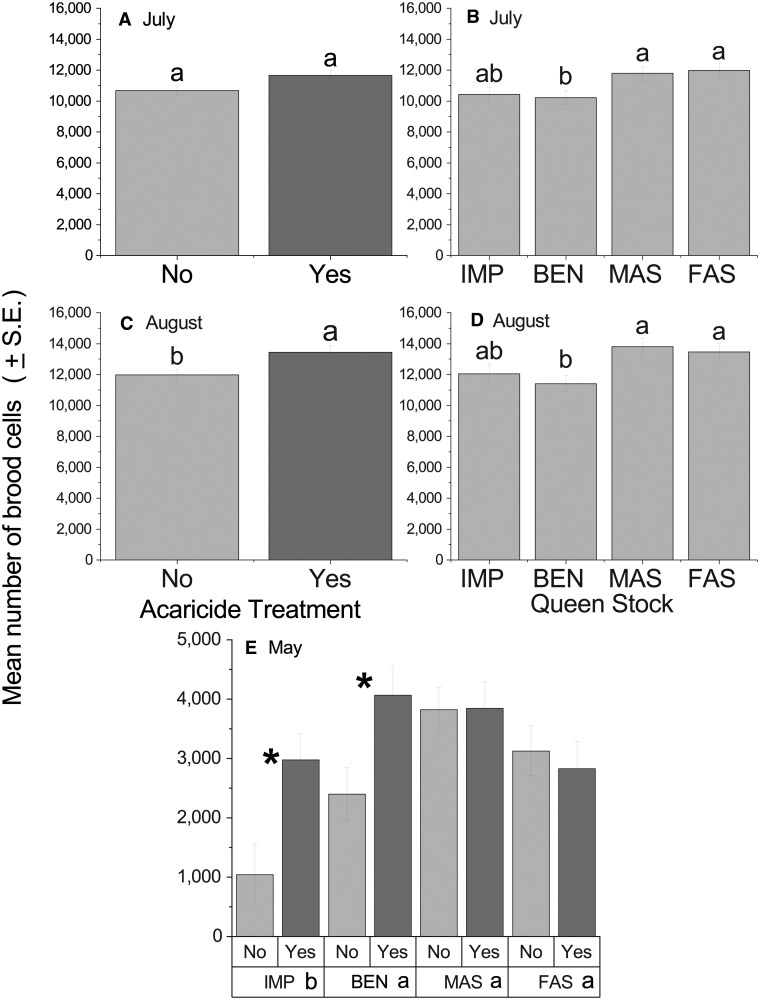


### Adult Bees

**Fig. 2.**
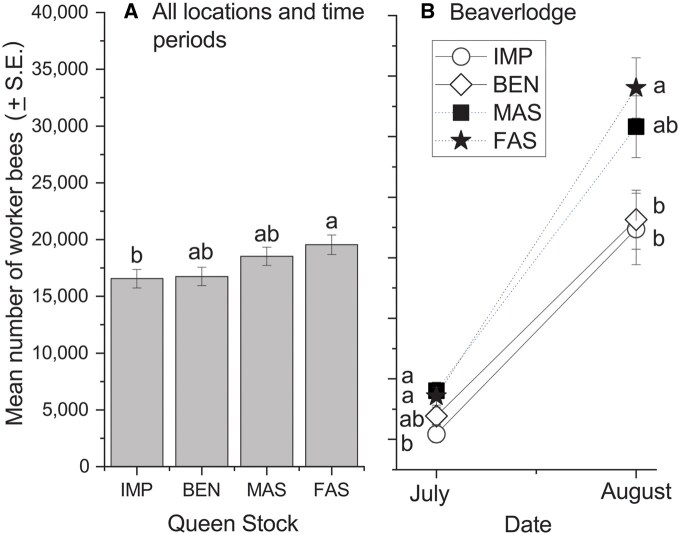
Effect of stock on worker population size (mean number of worker bees ± SE) when A) averaged over all locations and dates (July, Aug 2013, and May 2014) and B) from July to August 2013 at the Beaverlodge site only. Stocks with the same letters within a date are not significantly different from each other (ANOVA; Tukey *P* < 0.05). Colony populations increased from July to August (ANOVA; Tukey P < 0.05). Abbreviations: BEN, Benchmark stock; FAS, Field Assisted Selection; IMP, Imported; MAS, Marker Assisted Selection.

Worker populations were higher in FAS colonies than in IMP colonies, while MAS colonies did not differ from FAS, BEN, or IMP stock (Fig. 2A; *F *= 2.97; df = 3, 187; *P *= 0.03). All colonies grew between July and August (*F *= 464.96; df = 1, 178; *P *< 0.0001) with the relative differences among stocks averaged over all sites remaining consistent over time (*F *= 0.13; df = 3, 178; *P *= 0.95). However, there was a significant location × stock × date interaction (*F *= 2.35; df =6, 178; *P *= 0.03), driven primarily by the Beaverlodge location. At Beaverlodge, stock effects were pronounced in both July (F = 4.72; df = 3, 198; P = 0.003) and August (F = 8.60; df = 3, 185; P < 0.0001), whereas at Manitoba and Lethbridge no significant differences were observed (*P* > 0.05). Detailed analysis at Beaverlodge revealed that stock differences changed over time (*F* = 5.85; df = 3, 71.6; *P* = 0.0012): in July, FAS and MAS colonies had larger worker populations than IMP, with BEN intermediate; by August, FAS colonies exceeded both IMP and BEN, while MAS remained intermediate and did not differ from any other stock (Fig. 2B).

### Fall and Spring Cluster Size and Winter Survival

**Fig. 3.**
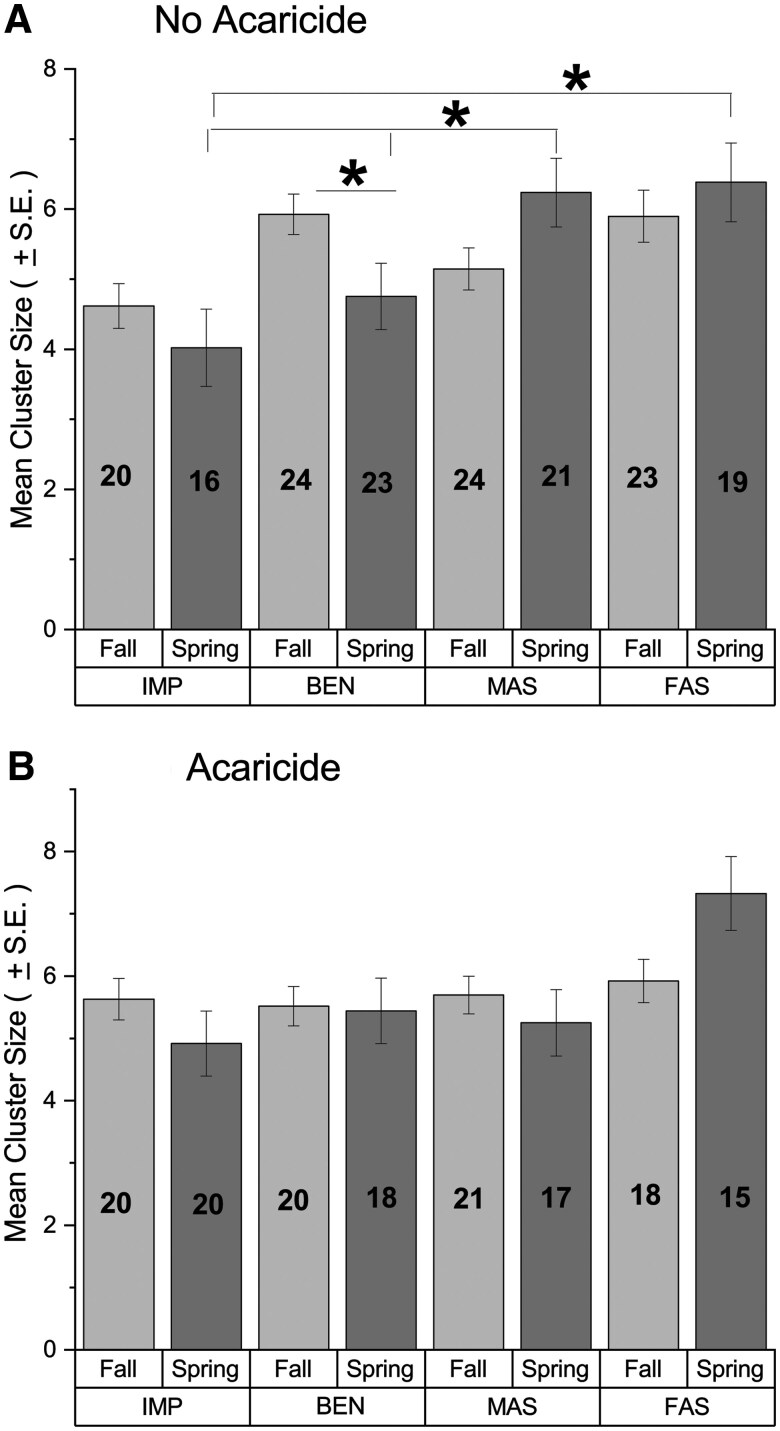
Effects of acaricide treatment, stock, and season on size of worker honey bee clusters (# of inter-frame spaces filled with bees ± SE) prior to wintering in late fall 2013 and in early spring 2014 following removal from indoor wintering facilities. Data are pooled for Beaverlodge AB, Lethbridge, AB, and Winnipeg, MB. Horizontal lines with asterisks represent significant differences among queen stocks within each acaricide treatment (*P* < 0.05, protected contrasts). Bold numbers represent sample size. Abbreviations: BEN, Benchmark stock; FAS, Field Assisted Selection; IMP, Imported; MAS, Marker Assisted Selection.

The size of the cluster of adult worker bees was assessed in late fall before bees were moved into their winter storage buildings and immediately after they were moved outside the following spring. There were significant overall effects of stock on cluster size (*F *= 5.09; df = 3, 156; *P *= 0.002), but there was also a significant interaction between acaricide treatment, stock, and season (*F *= 3.21; df = 3, 156; *P *= 0.025). Similar to the pattern observed for brood production, there were significant effects of stock with season among the treatment groups that did not receive acaricide treatment (*F *= 4.35; df = 7, 156; *P *= 0.0002, Fig. 3A) but no significant differences among those that did receive treatment (*F *= 1.46; df = 7, 156; *P *= 0.19, Fig. 3B). Within the group of colonies that were untreated, cluster size declined significantly over winter in the BEN stock but not in any of the other stocks (Fig. 3A). MAS selected colonies had larger spring cluster sizes than either BEN or IMP colonies and FAS had larger spring cluster sizes than IMP stock (Fig. 3A). Colony survival from fall to spring was not affected by acaricide treatment (df = 1, χ^2^ = 0.23; *P* = 0.6342) or stock (df = 3, χ^2^ = 2.48; *P* = 0.48) nor was there any interaction between acaricide treatment and stock (df = 3, χ^2^ = 0.90; *P* = 0.82). Overall, 84% (176/210) of introduced marked queens remained at the time of the August assessment, and 74% (155/210) survived until late fall with roughly equal proportions in each stock. Eighty eight percent of colonies with original queens still present in fall in October that were treated with acaricide, and 87% of untreated colonies survived until spring. Overall, winter survival of colonies headed by IMP queens was 97% (30/31 original queens in October), BEN 88% (36/41), MAS 88% (30/34), and FAS 88% (30/34).

### Fall Weight

The weight of colonies that were assessed just prior to being wintered varied with location (*F *= 68.48; df = 3, 119; *P *< 0.0001) and stock (*F *= 2.85; df = 3, 119; *P *= 0.04) but was not affected by the spring acaricide treatment (*F *= 1.48; df = 1, 119; *P *> 0.23). Fall colony weights at all locations differed from one another (*P *< 0.05; Tukey) and were greatest in Lethbridge (42.9 ± 0.5 kg), intermediate in Beaverlodge (39.1 ± 0.4 kg), and lowest in Manitoba (36.0 ± 0.4 kg). Colonies of the IMP stock weighed 1.7 kg more than those of the MAS stock (*P *< 0.05; Tukey), but no other differences among fall colony weights were observed among the stocks (IMP 40.8 ± 0.5; BEN 38.9 ± 0.4; MAS 38.9 ± 0.4; FAS 38.9 ± 0.5 kg/colony).

### Weight Loss Over Winter

**Fig. 4.**
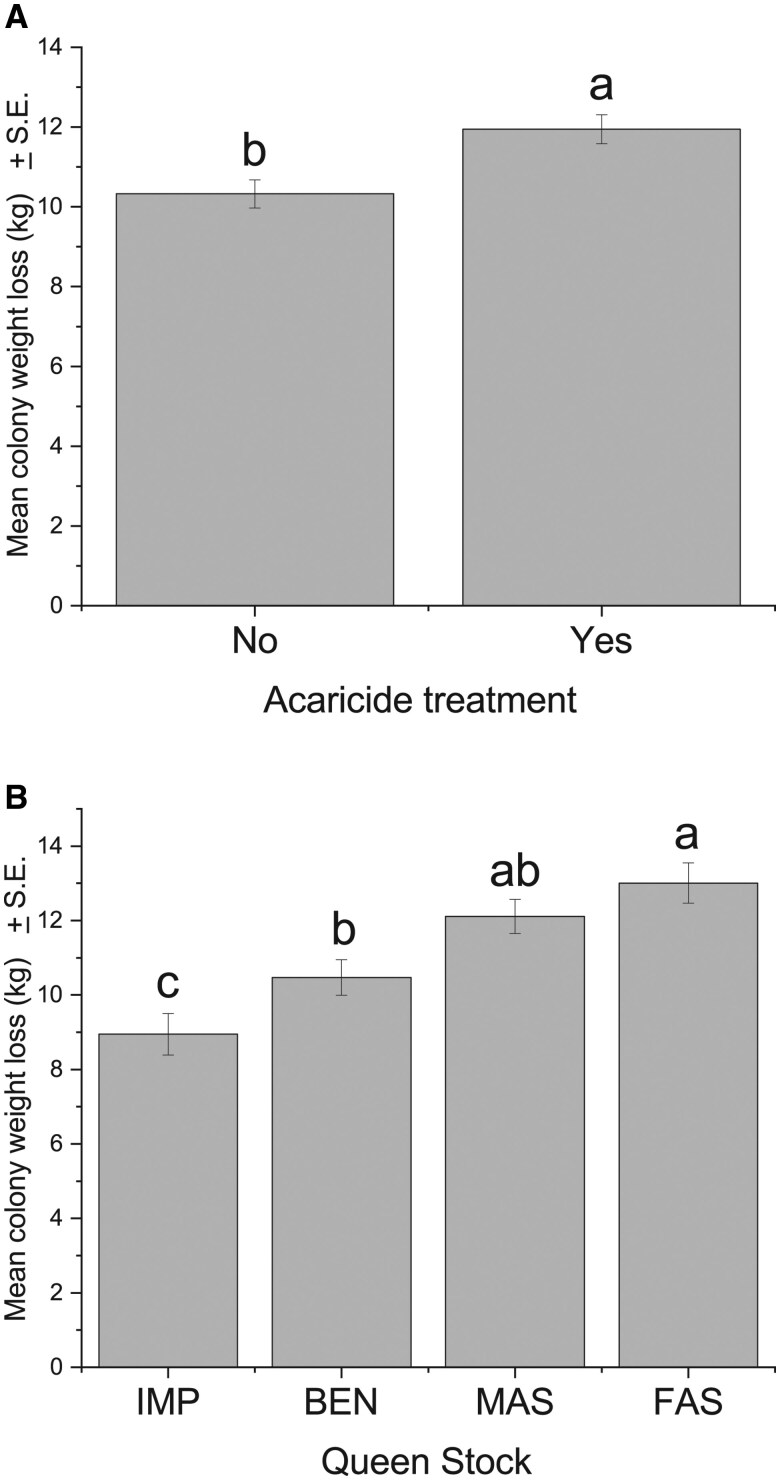
Effects of acaricide treatment A) and stock B) on colony weight loss over winter from the time colonies were moved into indoor wintering facilities in late fall until their removal the following spring. Bars with the same letter above them within each graph are not significantly different from each other (ANOVA; *P* < 0.05; Tukey). Abbreviations: BEN, Benchmark stock; FAS, Field Assisted Selection; IMP, Imported; MAS, Marker Assisted Selection.

Averaged across sites (within location), colony weight loss differed significantly by location (*F *= 23.10; df = 2, 102; *P *< 0.0001), acaricide treatment (*F *= 10.68; df = 1, 102; *P *= 0.002, Fig. 4A), and stock (*F *= 11.87; df = 3, 102; *P *< 0.0001, Fig. 4B). Colonies wintered in Beaverlodge and Manitoba lost similar amounts of weight (*P *> 0.05; Tukey), and both lost more than those in Lethbridge (P < 0.05; Tukey). Across locations, MAS, FAS, and BEN colonies lost more weight than IMP colonies, and FAS colonies lost more weight than BEN colonies (*P *< 0.05; Tukey), while MAS and FAS colonies did not differ (*P *> 0.05; Tukey) (Fig. 4B).

Although 2-way interactions among stock, location, and acaricide on weight loss were not significant (*P *> 0.05), a significant 3-way interaction was detected (location × acaricide × stock) (*F *= 2.37; df = 6, 102; *P *= 0.04). Partitioning the 3-way interaction by location indicated that strongest interactions among acaricide and stock were at the Manitoba (*F *= 7.14; df = 7, 102; *P *< 0.0001) and Lethbridge locations (*F *= 3.45; df = 7, 102; *P *= 0.002) with no significant interaction at Beaverlodge (*F *= 1.83; df = 7, 102; *P *= 0.09). In Manitoba, stock differences were evident both with (*F *= 11.46 df = 7, 102; *P *= 0.0001) and without (*F *= 4.10; df = 7, 102; *P *= 0.009) acaricide treatment. In Lethbridge, stock differences occurred only in treated colonies (*F *= 4.13; df = 7, 102; *P *= 0.008; Slice) and not in untreated colonies (*F *= 1.61; df = 7, 102; *P *= 0.19; Slice). In both Manitoba and Lethbridge, MAS colonies lost more weight when treated with acaricide than when untreated (Lethbridge unexposed, 7.7 ± 1.2 vs exposed 12.8 ± 1.2; Manitoba untreated 10.9 ± 1.0 vs treated 16.3 ± 1.1 kg/colony). In contrast, weight loss in all other stocks at these locations did not differ with acaricide treatment (*P *> 0.05; Slice).

### Honey Production

Neither stock (*F *= 1.60; df = 3, 151; *P *= 0.19) nor acaricide treatment (*F *= 0.06; df = 1, 151; *P *= 0.43) affected honey production, and there were no significant 2- or 3-way interactions between acaricide treatment, stock, and apiary site (*P *> 0.05). However, there were significant differences in honey production among apiary sites. Average honey production across all sites and treatments was 67.4 ± 2.5 kg per hive. Production in the 2 Manitoba apiary sites, Glenlea 1 and 2, averaged 68.2 ± 4.9 and 87.2 ± 4.6 kg per colony, respectively; at the 2 Beaverlodge sites, production averaged 67.5 ± 5.5 and 77.9 ± 5.6 kg per colony, respectively; and at the Lethbridge location, production averaged 37.7 ± 4.8 kg per colony. Production was significantly greater in the Glenlea 1, Glenlea 2, and Beaverlodge 1 apiaries than in Lethbridge (*P *< 0.05, Tukey). The Glenlea 1 site also produced more honey than Beaverlodge 2 (*P *< 0.05, Tukey). All other differences among apiary sites were not significant. (*P *> 0.05, Tukey).

### Hygienic Behavior

**Fig. 5.**
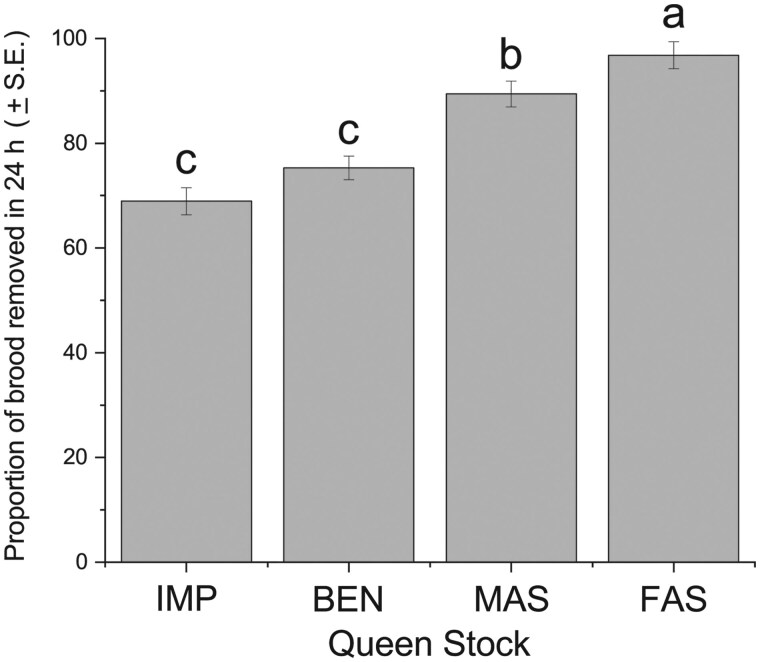
Effect of 4 different stocks on hygienic behavior (proportion of frozen brood cells completely removed within 24 h after exposure to freezing with liquid nitrogen ± SE). Bars followed by the same letter are not significantly different from each (ANOVA; Tukey *P* < 0.05). Abbreviations: BEN, Benchmark stock; FAS, Field Assisted Selection; IMP, Imported; MAS, Marker Assisted Selection.

Hygienic brood removal behavior was affected by stock (Fig. 5, F = 34.96; df = 3, 151; *P *= 0.0001) and location (*F *= 9.19; df = 2, 151; *P *= 0.0002) but not acaricide treatment (*F *= 0.53; df = 1, 151; *P *= 0.47). There were no significant interactions among location and stock or location and acaricide treatment (*P *> 0.05). When averaged across all sites, stock from both MAS (89.41 ± 2.5% removal) and FAS (96.8 ± 2.6%) selected queens had significantly higher freeze-killed brood removal than did the BEN (75.3 ± 2.3%) or IMP stocks (68.9 ± 2.6%) (*P *< 0.0001; Tukey) (Fig. 5). While the FAS-selected colonies had a higher proportion of brood removed than the MAS colonies (*P *< 0.02; Tukey), the rate of removal between the BEN and IMP was similar (*P *> 0.05; Tukey). When averaged across all apiaries, Beaverlodge had significantly greater levels of brood removal (86.9 ± 1.9%) than Manitoba (76.5 ± 1.8%), while Lethbridge (84.4 ± 2.6%) was intermediate (*P *> 0.05; Tukey).

### Parasites and Pathogens

#### Varroa

**Fig. 6.**
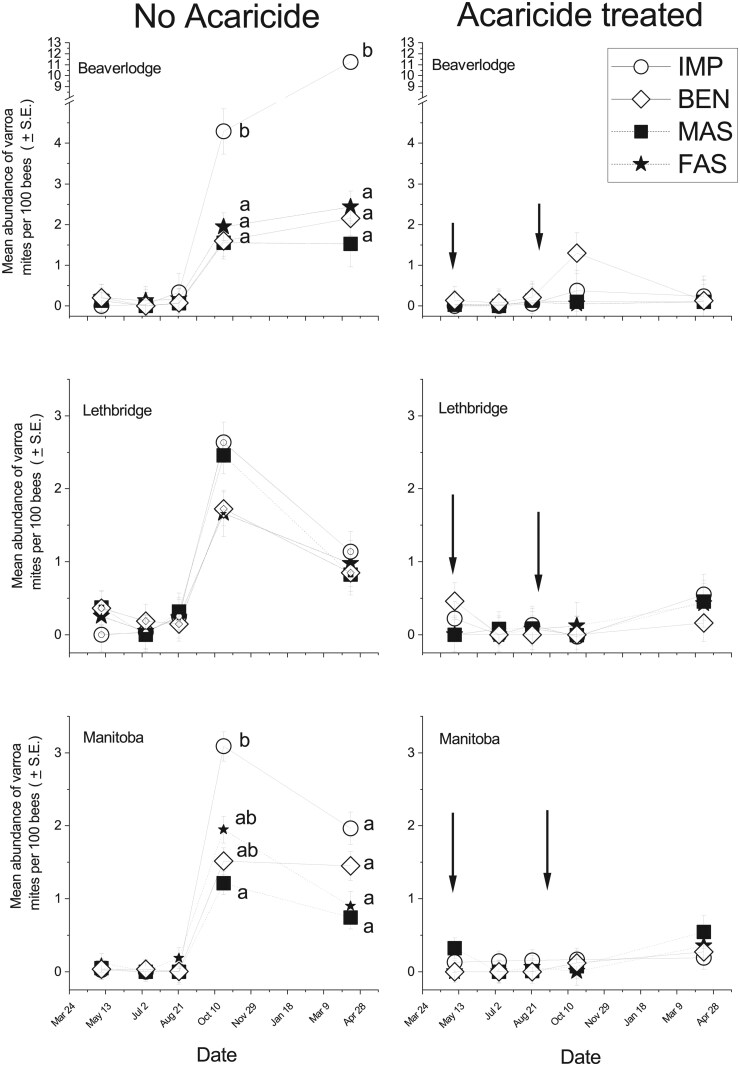
Effect of stock over time on the mean abundance of *Varroa* (mites per 100 bees ± SE) in colonies that were left untreated in spring and fall or received an acaricide (Apivar) for all locations. *Varroa* levels varied significantly between stocks in the absence of acaricide treatment (depending on location) but were similar among all sites when treated with acaricide. Means followed by the same letter within an assessment date are not significantly different from each other (*P* < 0.05). Arrows indicate the timing of acaricide treatments. Abbreviations: BEN, Benchmark stock; FAS, Field Assisted Selection; IMP, Imported; MAS, Marker Assisted Selection.

When all sites were pooled for analysis of mean *Varroa* abundance, the effects of stock, acaricide treatment, assessment date, and location as well as all possible interactions between these factors were significant (*P *< 0.05) (Fig. 6). There was a significant 4-way interaction between location, stock, acaricide treatment and assessment date (*F *= 1.65; df = 24, 548; *P *= 0.027). Contrasts to look at the combined effects of location and assessment date on the stock × acaricide × date × location interaction showed that significant effects related to stock and acaricide treatment were found only in Oct 2013 and May 2014 at the Beaverlodge and Manitoba locations and only in Oct 2013 at the Lethbridge location (*P *< 0.0001, Slice) (Fig. 6). Separate analyses by site indicated significant differences in the stock × acaricide × date for mean *Varroa* abundance at Beaverlodge (*F *= 3.57; df = 12, 211; *P *= 0.0001), and Manitoba (*F *= 2.20; df = 12,214; *P *= 0.013) but not at Lethbridge (*F *= 1.30; df = 12,123; *P *= 0.23), where no significant differences among stocks or interactions between stock and other factors were found (*P *> 0.05). When colonies were treated with acaricide, there were no differences in mean abundance of *Varroa* at any location (*P *> 0.05). When not treated with acaricide, at Beaverlodge the IMP stock had significantly greater mean *Varroa* abundance than the FAS, MAS or BEN stock in Oct 2013 and May 2014 (*P *< 0.05, Tukey, Fig. 6). For Manitoba, the IMP stock and BEN stock both had significantly greater mean *Varroa* abundance than the MAS stock in Oct 2013 and significantly greater mean *Varroa* abundance than the FAS or MAS by May 2014 (*P *< 0.05, Tukey, Fig. 6). At Lethbridge, there were no significant differences among stocks at any assessment date (*P *< 0.05, Tukey).

#### American Foulbrood

The average number of detectable *P. larvae* colony forming units found in adult bees (CFU/bee) was very low across all sampling dates and sites, but did vary among the 3 locations (Lethbridge AB, 0 ± 0.0; Beaverlodge AB, 1.1 ± 278; and Glenlea MB, 507 ± 269 CFU/bee) (*F *= 130.5; df = 2,198; *P *= 0.0001). Because Lethbridge colonies had no detectable American foulbrood spores, the location was excluded from further analysis. The 2 locations with detectable American foulbrood spores on adult bees (Beaverlodge 22/67 colonies, Manitoba 74/83 colonies) showed patterns of variation with assessment date (*F *= 59.7; df = 3, 352; *P *= 0.0001) that differed between apiary sites (*F *= 200.4; df = 3, 160; *P *= 0.0001) but the seasonal patterns were not influenced by either main effect treatment. There was, however, a significant 3-way interaction between stock, acaricide treatment and assessment date (*F *= 3.11; df = 3,160; *P *= 0.028, Supplementary Fig. S1). Partitioning this interaction by location showed that there was no interaction between stock and acaricide at the Beaverlodge location (*F *= 0.36; df = 7, 160; *P *> 0.92) while there was an interaction at the Manitoba location, which also had higher levels (*F *= 3.19; df = 3, 77; *P *= 0.02). There was an overall effect of stock on American foulbrood within Manitoba (*F *= 4.59; df = 7, 160; *P *= 0.0001) with the FAS colonies having higher levels of American foulbrood than the IMP colonies (Supplementary Fig. S1). Partitioning the stock by acaricide treatment interaction showed no effect of stock when colonies were treated with acaricide (*F *= 0.05; df = 3,77; *P *= 0.98), but differences in American foulbrood levels among stocks were noted in the absence of spring acaricide treatment (*F *= 6.84; df = 3, 77; *P *= 0.0006). Within untreated colonies, the FAS stock had higher American foulbrood levels than the IMP stock (*P *< 0.05, Tukey), and within the FAS stock, untreated hives had higher levels of American foulbrood than the treated hives (*P *< 0.05, Slice). Within the Beaverlodge location there was also a date × acaricide interaction (*F *= 3.90 df = 3, 90; *P *= 0.01) with treated colonies having slightly greater CFUs (4.3 ± 0.8) than untreated colonies (1.4 ± 0.7) but this difference was only significant in Oct 2013 (*F *= 11.8; df = 3,77; *P *= 0.0008; Slice).

#### Vairimorpha

Mean abundance of *Vairimorpha* spores was not affected by stock (*F *= 0.28; df = 3, 198; *P* = 0.84) or acaricide treatment (*F *= 0.11; df = 1, 198; *P *= 0.73), nor were there any significant interactions between the main effects and assessment date or location (*P *> 0.05). Although there was a significant interaction between apiary site and assessment date (Supplementary Fig. S2, *F *= 10.27; df = 6, 329; *P *= 0.0001) with the 3 locations having different levels on most assessment dates, the overall mean abundance of *Vairimorpha* did not differ among locations (*F *= 0.44; df = 1,198; *P *= 0.64). Mean abundance of *Vairimorpha* averaged 281,520 ± 117,113; 110,548 ± 187,783; and 453,868 ± 104,286 spores per bee in Beaverlodge, Lethbridge, and Manitoba, respectively.

The prevalence of colonies infected with either *V. apis, V. ceranae* or mixes of both spp., varied with assessment date in all 3 locations (Beaverlodge; df = 3; χ^2^ = 64.8; *P* = 0.001; Lethbridge df = 2; χ^2^ = 19.05, *P *= 0.001; Manitoba df = 3; χ^2^= 38.07; *P *= 0.001) but was not affected by stock (*P *> 0.05; χ^2^) or acaricide treatment (*P *> 0.05; χ^2^) (Supplementary Fig. S2). All 3 locations showed a similar pattern of change, with *Vairimorpha* prevalence declining slightly in mid-summer and increasing in the fall. There was a significant shift in the relative proportion of colonies infested with each *Vairimorpha* species over the duration of the experiment (Supplementary Fig. S2). The proportion of colonies found with pure *V. ceranae* increased over time at each of the 3 locations (Beaverlodge: df = 3; χ^2^ = 32.0; *P *= 0.001; Lethbridge: df = 2; χ^2^ = 40.9; *P *= 0.0001; Manitoba: df = 3; χ^2^ = 10.52; *P *= 0.015). At the Beaverlodge location, acaricide-treated colonies had a lower overall proportion of colonies affected with *V. ceranae* (df = 1; χ^2^ = 3.98, *P *= 0.046), but the interaction between acaricide and stock was not significant (df = 3; χ^2^ = 2.03; *P *> 0.57). Acaricide treatment did not affect the proportion of colonies with *V. ceranae* in Manitoba (df = 1; χ^2^ = 1.02; *P *> 0.31) or Lethbridge (df = 1; χ^2^ = 0.12; *P *> 0.77).

#### Visible Symptoms of Brood Diseases and Deformed Wing Virus

Prevalence of chalkbrood infection in colonies varied between assessment dates (df = 2, χ^2^ = 26; *P *< 0.0001), with the greatest prevalence in July 2013 after colonies had been initiated from New Zealand package bees (Table 1). Differences among stocks were apparent only in May of the second year (*P *< 0.003, Fisher’s exact test); IMP colonies had a greater prevalence of chalkbrood than BEN but did not differ from FAS or MAS (Table 1). Prevalence of all other pathogens was very low and did not differ among stocks. Only 3 colonies with symptoms consistent with sacbrood virus were found in July 2013 (2 IMP and 1 BEN; *P *> 0.32) and 2 more in May 2014 (2 IMP; *P *> 0.71). Finally, only 1 colony was detected with visible DWV (BEN; *P *> 0.49). No visible symptoms of American foulbrood or European foulbrood were apparent across the entirety of this study.

**Table 1.** Prevalence of chalkbrood in colonies headed by 4 different stocks throughout the trial

Assessment date	IMP	BEN	MAS	FAS	*P*
**July 2013**	21.2% (*n* = 52)	20.4% (*n* = 54)	25.5% (*n* = 55)	17.3% (*n* = 52)	0.786
**Aug 2013**	2.6% (*n* = 39)	6.5% (*n* = 46)	7.1% (*n* = 42)	4.5% (*n* = 45)	0.853
**May 2014**	14.8% (*n* = 27)a	0.0% (*n* = 33)b	3.2% (*n* = 31)ab	0.0% (*n* = 27)ab	0.015

Prevalence was defined by the % of colonies with visible symptoms.

Means followed by the same letter within assessment dates are not significantly different (Fisher’s exact test).

Abbreviations: BEN= Benchmark; FAS= Field-Assisted Selection; IMP, Imported; MAS, Marker-Assisted Selection.

## Discussion

Selecting and propagating stocks for economically important traits is central to livestock management, including beekeeping systems that use integrated pest management to support colony health. In this study, queen bees were selectively bred for hygienic brood removal, a form of social immunity, using either a standard field-based phenotypic assay (FAS) or protein marker-assisted selection (MAS) (Guarna et al. 2017). Both methods significantly improved parasite management when compared with unselected domestic stocks (BEN) or with commercially imported (IMP) stocks. Importantly, hygienic behavior imposed no detectable biological or productivity costs, regardless of the selection method used, even under conditions of minimal pathogen stress across multiple field sites.

### Worker Brood, Adult Population, and Honey Production

The ability of a honey bee queen to produce brood and expand the colony population is directly linked to colony health, productivity, and fitness (Winston 1987, Hernandez et al. 2023). Colonies with strong populations are desirable for both honey production and pollination services. There could be a potential cost of hygienic behavior if colonies expressing this trait removed a greater proportion of healthy brood than unhygienic colonies, in addition to diseased brood. However, previous studies have found that colonies with high hygienic behavior have similar worker adult and brood production to unhygienic colonies (Spivak and Reuter 2001a), and the removal of dead and healthy brood does not appear to be correlated (Bigio et al. 2014). In this study, colonies headed by MAS and FAS queens generally produced more brood and maintained larger populations than colonies headed by domestic benchmark queens or unselected imported queens, although effects varied over time.

In this study, selectively bred disease-resistant colonies (MAS and FAS) produced the same amount of honey as the unselected colonies, indicating no productivity costs associated with the enhancement of hygienic behavior. Honey production is a key economic trait, and previous studies have similarly found that colonies that exhibit hygienic behavior have equivalent (Strange and Calderone 2009, Bixby et al. 2017, Guarna et al. 2017), or higher honey production (Spivak and Reuter 1998, 2001b, Garcia et al. 2013) than non-hygienic ones. However, many comparisons use stocks from different genetic backgrounds, limiting conclusions about trade-offs (Spivak and Reuter 1998, Leclercq et al. 2017). A strength of our study is that the BEN and hygienic stocks were drawn from the same base population and bred over the same number of generations. We also had robust sample sizes and colonies tested across multiple highly productive regions for honey production, where the sensitivity to detect differences in production should be high. Under these conditions, we not only saw no increase in honey production associated with selection for hygienic behavior but also saw no evidence of costs, even in conditions where *Varroa* were effectively controlled to minimize confounding effects that could result from parasitism pressure.

Overwintering colony weight loss results require a careful interpretation. Colonies headed by vigorous queens with healthy bees typically overwinter with larger populations (Harris 2008), they store more resources in fall (Richardson et al. 2023), and produce more brood over the winter, but also consume more stored food. As a result, greater winter weight loss is expected in larger colonies. Because feed costs are a major economic consideration in temperate beekeeping, overwinter feed use is an important consideration in selection programs, particularly when comparing colonies of similar size. In this study, colonies headed by MAS and FAS queens lost more weight over winter than IMP colonies, likely due to higher feed demands from larger worker populations. Similarly, acaricide-treated colonies lost more weight than untreated colonies, consistent with their larger populations and greater winter brood production. Although larger colonies require more feed, they also have higher overwinter survival and provide greater honey production and pollination capacity in spring, offsetting these additional costs with both increased biological fitness and economic productivity.

### Varroa

Colonies exhibiting hygienic behavior have been found to reduce *Varroa* abundance, at least to some degree, for as long as one year (Ibrahim et al. 2007, Spivak and Reuter 2001b). However, while there is consensus in the literature that hygienic behavior is effective against American foulbrood and chalkbrood, the relationship between hygienic behavior and control of the *Varroa* mite appears to be more complex (reviewed in LeClercq et al. 2017). In this study, *Varroa* levels were generally low, which likely explains the absence of stock effects at the Lethbridge site. When colonies received effective mite treatments, no differences in *Varroa* infestation were observed among stocks. In contrast, under higher parasite pressure at Beaverlodge and Manitoba, IMP and unselected BEN stocks had higher levels than MAS colonies after 4 mo, and higher levels than MAS or FAS colonies after one year. At Beaverlodge, the IMP stock had consistently greater *Varroa* abundance than the FAS, MAS, or BEN stock at both timepoints. Further, although untreated MAS and FAS colonies had higher mite levels than treated counterparts, they performed similarly in key health and economic metrics, including population size and honey production, with benefits persisting one year after establishment. Whereas the genetics of MAS, FAS, and BEN stock originated in Canada, the queens of IMP stock came from New Zealand, where the beekeeping conditions are very different from Canada, and *Varroa* has been more recently introduced. Likewise, the IMP stock also had higher levels of chalkbrood than the BEN stock after one year. These differences illustrate the value of queens produced regionally, and how they may support integrated pest management of *Varroa* mites and other pathogens, even without selective breeding.

Colonies bred for *Varroa*-sensitive hygiene have been shown to reduce *Varroa* abundance by an average of 44% (range: 22% to 74%) (Danka et al. 2011) delay the time required for mite populations to reach treatment thresholds by approximately 20% (≈12.5 wk) (Delaplane et al. 2005), and reduce the need for acaricide treatments (Ward et al. 2008). In this study, the FAS stock was not selected for Varroa-sensitive hygiene, and only 4 of 13 protein markers used in the MAS stock were associated with this trait. Despite this, both MAS and FAS stocks exhibited significantly lower mean Varroa abundance than IMP stock. An economic analysis of MAS stock from the same breeding program demonstrated substantial financial benefits, particularly under conditions of reduced acaricide efficacy or emerging resistance (Bixby et al. 2017). Although the acaricide used in this study (Apivar, active ingredient amitraz) was effective at the time, subsequent research has documented widespread amitraz resistance (Rinkevich 2020, Bahreini et al. 2025), further emphasizing the value of behaviorally disease-resistant stocks.

While we did not quantify viruses in this study, it is well-established that bee viruses are highly correlated with *Varroa* mite levels, as *Varroa* mites serve as vectors of many viruses (Le Conte et al. 2010, Di Prisco et al. 2016, Doublet et al. 2024). In this study, visible symptoms of sacbrood and deformed wing virus were rare; however, no viruses were assessed with molecular analysis. Previous studies have reported lower levels of *Varroa*-associated viruses, including DWV-A, DWV-B, and CBPV, in *Varroa*-resistant stocks such as “Pol-line” bees (O’Shea‑Wheller et al. 2022, Borba et al. 2022). The lower mean Varroa abundance observed in untreated FAS and MAS colonies compared with BEN and IMP stocks suggests these stocks may also reduce virus risk, although this requires further investigation.

### Other Pathogens

These results are consistent with our previous findings (Guarna et al. 2017), in which both FAS and MAS stocks exhibited significantly higher hygienic behavior than unselected stocks. This study extends that work by evaluating stocks across multiple geographic regions, under contrasting Varroa management regimes (acaricide-treated versus untreated), and after 3 generations of selection. Importantly, it directly compares stocks selected for hygienic behavior over 3 generations with unselected stocks derived from the same original breeding population and maintained for the same number of generations, allowing a direct assessment of potential costs associated with resistance. In contrast to Guarna et al. (2017), in which the stocks were challenged with high levels of American foulbrood-contaminated comb, MAS and FAS stocks here did not significantly reduce American foulbrood spore loads or chalkbrood levels, likely due to very low pathogen presence and absence of clinical symptoms. Previous studies have demonstrated benefits of hygienic behavior against American foulbrood, chalkbrood, and *Varroa* mites under higher pathogen or parasite burden (Spivak and Gilliam 1998).

We observed significant interactions between stock and acaricide treatment for American foulbrood spore levels at the Manitoba and Beaverlodge locations. Although it is unclear why FAS colonies selected for hygienic behavior would exhibit statistically higher American foulbrood levels than the unselected stock (IMP) only at the MB site, contrary to previous studies (eg Spivak and Reuter 2001a, Guarna et al. 2017), it is important to note that only spore loads on adult bees were measured, and no clinical symptoms were observed. Hygienic colonies may contain low levels of spores while effectively preventing disease expression, particularly when brood or stored resources are lightly contaminated. Moreover, spore loads in this study were frequently non-detectable, and even when they could be detected, they were far below those associated with clinical disease (>3,000 spores per bee; Gende et al. 2011). This suggests that our statistically significant interaction is unlikely to be biologically relevant. Based on the comparatively low *P. larvae* spore abundance detected within bees across all locations, and the long-term culturing of *P. larvae* across multiple inspection dates, the potential confound with having Manitoba colonies treated with oxytetracycline at establishment does not compromise our overall interpretations related to stock-mediated disease resistance in this study. In addition, the absence of consistent differences in AFB levels between hygienic and BEN stocks further indicates no evidence of biological trade-offs associated with selection for hygienic behavior. Nevertheless, these findings underscore the value of multi-trait selection and support the inclusion of additional performance and health traits in future marker panels to ensure that gains in hygienic behavior are achieved without unintended consequences for other aspects of colony performance.

Holmes et al. (2023) found that colonies headed by New Zealand queens are associated with higher chalkbrood levels in the study region. In this study, all colonies in this study were started from NZ packages and showed symptoms of chalkbrood 2 months after establishment in July 2013, but only the IMP stock (with New Zealand queens) maintained higher chalkbrood levels through to May 2014. It is likely that the chalkbrood levels in this study were driven by the New Zealand origin of the package bees, as all domestic stocks (BEN, MAS, and FAS) reduced chalkbrood in the second year without any associated costs, demonstrating effective resistance.

*Vairimorpha* infections primarily manifest in older adult bees; therefore, hygienic behavior assays that evaluate colony responses to dead brood would not be predicted to be effective for selecting for resistance to *Vairimorpha* or other pathogens that do not target brood. Although some breeding programs for hygienic behavior have been linked to overall reductions in *Vairimorpha* at the apiary level (Ivgin Tunca et al. 2017), there is no evidence that hygienic behavior directly reduces *Vairimorpha* loads or that it is influenced by spore levels (Valizadeh et al. 2020). The UBeeO assay was developed to measure olfactory sensitivity and responsiveness to brood pheromones, and while it likely has some overlap with traits involved in hygienic behavior, it does not select for hygienic behavior in the strict traditional sense. This distinction may help explain why UBeeO selection has been associated with reduced *Vairimorpha* loads, but not prevalence, in adult bees (Alger et al. 2025). In this study, the prevalence of *Vairimorpha* generally increased in the autumn, underscoring the importance of managing this disease to protect the developing winter bees, particularly under compounding stressors such as *Varroa* mite infestations. The observed shift toward a higher proportion of *V. ceranae* relative to *V. apis* at all 3 sites is consistent with previous findings by Huang et al. (2015). We found no benefits of hygienic behavior, nor any costs or trade-offs associated with *Vairimorpha* defense; however, these could have been masked by treatment with Fumagilin-B (Peirson and Pernal 2024).

### Breeding Considerations

The cost of testing and propagating beneficial traits in a selection program can be significant. Large queen breeding operations must screen hundreds to thousands of colonies to provide sufficient colonies for producing queens and drones with favourable genetics. Methods that improve selection efficiency allow breeders to screen more colonies and increase the availability of highly selected queens. However, selection programs must also consider potential trade-offs associated with emphasizing a single trait. Although MAS and FAS stocks performed similarly in most cases in this study, future marker panels could incorporate multiple traits to ensure that gains in hygienic behavior are achieved without compromising other important aspects of colony performance. It is encouraging that MAS performed equivalently to the FAS stock, and we would expect that further marker refinement could benefit the selection process and increase gains in selected traits. The MAS technique provides a more rapid and less laborious selection method compared to FAS, which could be particularly advantageous for large queen breeding operations, and an ongoing benefit of MAS is that as additional markers are identified different traits could be added in on the same test at little extra expense (Bixby et al. 2017).

## Conclusion

This study demonstrates that selecting honey bee stocks for disease resistance using either marker-assisted or field -assisted selection offers significant benefits compared to unselected stocks, without compromising key productivity traits such as brood production, adult bee population, honey yield, or winter survival. MAS stocks performed comparably to FAS stocks, highlighting the potential for MAS to increase the efficiency of queen breeding programs and make highly selected bees more widely available to beekeepers. This form of social immunity delivered clear gains in multiple indicators of colony fitness, with no evidence of significant biological costs or trade-offs that would undermine productivity or survival. Notably, selected stocks (MAS and FAS) maintained lower *Varroa* mite populations than unselected stocks (BEN and IMP) in the absence of acaricide treatment, demonstrating an enhanced capacity for natural mite suppression and reinforcing their value in integrated *Varroa* management.

## Supplementary Material

toag044_Supplementary_Data
