# Supplementary material for: Biological costs and benefits of selective breeding for disease resistance using marker-assisted or field-based selective breeding in honey bees (Apis mellifera)
Source: J Econ Entomol. 2026 Mar 3;119(3):1578–92. doi: 10.1093/jee/toag044 (PMC13365145; doi:10.1093/jee/toag044)
Supplement: toag044_Supplementary_Data [file toag044_supplementary_data.zip › toag044_Supplementary_Data/ECONENT-2025-0931 Supplement Revised.docx]

**Supplements**

**Supplemental Table 1.** Primers used for *Vairimorpha* spp. detection in multiplex end-point PCR

| **Assay** | **Primer** | **Sequence (5’ - 3’)** | **PCR product size (bp)** | **Reference** |
| --- | --- | --- | --- | --- |
| *V. ceranae* | 218MITOC-FOR | CGGCGACGATGTGATATGAAAATATTAA | 218 | Martín-Hernández  et al. (2007) |
|  | 218MITOC-REV | CCCGGTCATTCTCAAACAAAAAACCG |  |  |
|  |  |  |  |  |
| *V. apis* | 321APIS-FOR | GGGGGCATGTCTTTGACGTACTATGTA | 321 | Martín-Hernández  et al. (2007) |
|  | 321APIS-REV | GGGGGGCGTTTAAAATGTGAAACAACTATG |  |  |
|  |  |  |  |  |
| RpS5 | RpS5-For | AATTATTTGGTCGCTGGAATTG | 115 | Thompson  et al. (2007) |
|  | RpS5-Rev | TAACGTCCAGCAGAATGTGGTA |  |  |

|  | **Supplementary Table 2.** Number of colonies with original marked queens remaining in each group at each assessment date | | | | | | | | |
| --- | --- | --- | --- | --- | --- | --- | --- | --- | --- |
| **Site** | | **Apiary** | **Stock** | **Acaricide** | **May 2013** | **Jul 2013** | **Aug**  **2013** | **Oct**  **2013** | **Apr 2014** |
| Beaverlodge | | Yard 3 | IMP | no | 4 | 4 | 2 | 2 | 2 |
|  |  |  |  | yes | 6 | 6 | 4 | 4 | 4 |
|  |  |  | BEN | no | 6 | 6 | 4 | 3 | 3 |
|  |  |  |  | yes | 6 | 6 | 5 | 4 | 4 |
|  |  |  | FAS | no | 6 | 6 | 6 | 6 | 6 |
|  |  |  |  | yes | 6 | 6 | 6 | 3 | 3 |
|  |  |  | MAS | no | 6 | 6 | 4 | 3 | 2 |
|  |  |  |  | yes | 6 | 6 | 3 | 3 | 3 |
|  |  | Yard 4 | IMP | no | 6 | 6 | 4 | 2 | 2 |
|  |  |  |  | yes | 3 | 3 | 2 | 1 | 1 |
|  |  |  | BEN | no | 6 | 6 | 6 | 6 | 6 |
|  |  |  |  | yes | 5 | 5 | 3 | 3 | 2 |
|  |  |  | FAS | no | 5 | 5 | 5 | 4 | 3 |
|  |  |  |  | yes | 5 | 5 | 3 | 2 | 1 |
|  |  |  | MAS | no | 6 | 6 | 6 | 5 | 4 |
|  |  |  |  | yes | 4 | 4 | 4 | 4 | 4 |
| Lethbridge | | Barn Yard | IMP | no | 6 | 6 | 6 | 4 | 4 |
|  |  |  |  | yes | 6 | 6 | 5 | 5 | 5 |
|  |  |  | BEN | no | 6 | 6 | 6 | 6 | 5 |
|  |  |  |  | yes | 5 | 5 | 5 | 5 | 5 |
|  |  |  | FAS | no | 6 | 6 | 4 | 4 | 4 |
|  |  |  |  | yes | 6 | 5 | 4 | 3 | 3 |
|  |  |  | MAS | no | 6 | 6 | 4 | 5 | 5 |
|  |  |  |  | yes | 6 | 6 | 6 | 6 | 6 |
| Manitoba | | OBS | IMP | no | 5 | 5 | 5 | 3 | 3 |
|  |  |  |  | yes | 6 | 6 | 6 | 5 | 4 |
|  |  |  | BEN | no | 6 | 6 | 6 | 3 | 3 |
|  |  |  |  | yes | 5 | 5 | 5 | 5 | 5 |
|  |  |  | FAS | no | 5 | 5 | 6 | 3 | 3 |
|  |  |  |  | yes | 5 | 6 | 4 | 4 | 3 |
|  |  |  | MAS | no | 6 | 6 | 4 | 3 | 3 |
|  |  |  |  | yes | 5 | 5 | 3 | 3 | 2 |
|  |  | HOG | IMP | no | 6 | 6 | 2 | 2 | 2 |
|  |  |  |  | yes | 5 | 5 | 4 | 3 | 3 |
|  |  |  | BEN | no | 5 | 5 | 3 | 3 | 2 |
|  |  |  |  | yes | 4 | 5 | 3 | 2 | 2 |
|  |  |  | FAS | no | 7 | 7 | 5 | 3 | 2 |
|  |  |  |  | yes | 3 | 3 | 3 | 2 | 2 |
|  |  |  | MAS | no | 5 | 5 | 5 | 5 | 5 |
|  |  |  |  | yes | 5 | 5 | 4 | 4 | 2 |
| **Total** | |  |  |  | 214 | 214 | 175 | 146 | 133 |
